# Multilocus Sequence Typing and Virulence Potential of *Vibrio parahaemolyticus* Strains Isolated from Aquatic Bird Feces

**DOI:** 10.1128/spectrum.00886-22

**Published:** 2022-06-13

**Authors:** Chonchanok Muangnapoh, Eakapong Tamboon, Neunghatai Supha, Jirachaya Toyting, Atchara Chitrak, Nakarin Kitkumthorn, Peeraya Ekchariyawat, Tetsuya Iida, Orasa Suthienkul

**Affiliations:** a Department of Microbiology, Faculty of Science, Chulalongkorn University, Bangkok, Thailand; b Department of Microbiology, Faculty of Public Health, Mahidol University, Bangkok, Thailand; c Department of Oral Biology, Faculty of Dentistry, Mahidol University, Bangkok, Thailand; d Department of Bacterial Infections, Research Institute for Microbial Diseases, Osaka University, Suita, Osaka, Japan; Health Canada

**Keywords:** *Vibrio parahaemolyticus*, aquatic bird feces, multilocus sequence typing, virulence genes, whole-genome analysis

## Abstract

Vibrio parahaemolyticus is a Gram-negative, foodborne pathogenic bacterium that causes human gastroenteritis. This organism is ubiquitously present in the marine environment. Detection of V. parahaemolyticus in aquatic birds has been previously reported; however, the characterization of isolates of this bacterium recovered from these birds remains limited. The present study isolated and characterized V. parahaemolyticus from aquatic bird feces at the Bangpu Recreation Center (Samut Prakan province, Thailand) from 2016 to 2017, using multilocus sequence typing (MLST) and genome analysis. The results showed that V. parahaemolyticus was present in 34.9% (76/218) of the collected bird fecal samples. Among the *ldh*-positive V. parahaemolyticus isolates (*n* = 308), 1% **(**3/308**)** were positive for *tdh*, 1.3% **(**4/308**)** were positive for *trh*, and 0.3% **(**1/308**)** were positive for both *tdh* and *trh*. In turn, the MLST analysis revealed that 49 selected V. parahaemolyticus isolates resolved to 36 STs, 26 of which were novel (72.2%). Moreover, a total of 10 identified STs were identical to globally reported pathogenic strains (ST1309, ST1919, ST491, ST799, and ST2516) and environmental strains (ST1879, ST985, ST288, ST1925, and ST260). The genome analysis of isolates possessing *tdh* and/or *trh* (ST985, ST1923, ST1924, ST1929 and ST2516) demonstrated that the organization of the T3SS2α and T3SS2β genes in bird fecal isolates were almost identical to those of human clinical strains posing public health concerns of pathogen dissemination in the recreational area. The results of this study suggest that aquatic birds are natural reservoirs of new strains with high genetic diversity and are alternative sources of potentially pathogenic V. parahaemolyticus in the marine environment.

**IMPORTANCE** To our knowledge, infection of foodborne bacterium *V. parahamolyticus* occurs via the consumption of undercooked seafood contaminated with pathogenic strains. Aquatic bird is a neglectable source that can transmit *V. parahaemolyticus* along coastal areas. This study reported the detection of potentially pathogenic *V. parahamolyticus* harboring virulence genes from aquatic bird feces at the recreational center situated near the Gulf of Thailand. These strains shared identical genetic profile to the clinical isolates that previously reported in many countries. Furthermore, the strains from aquatic birds showed extremely high genetic diversity. Our research pointed out that the aquatic bird is possibly involved in the evolution of novel strains of *V. parahaemolyticus* and play a role in dissimilation of the potentially pathogenic strains across geographical distance.

## INTRODUCTION

Aquatic birds have been previously recognized as carriers of potentially pathogenic *Vibrio* species, including V. parahaemolyticus, V. cholerae, V. alginolyticus, V. campbellii, V. mimicus, V. vulnificus, and V. scophthalmi (1–7). During winter, a large number of aquatic birds usually migrate southward from their breeding colonies via transit across the Pacific Ocean (8–10). These migratory birds possibly play a vital role in the dissemination of *Vibrio* spp. in the coastal regions along their flyways (9–12). Among the *Vibrio* spp. commonly present in the marine environment, V. parahaemolyticus has been recognized as an important seafood-borne pathogen that causes human gastroenteritis and shrimp disease, the so-called acute hepatopancreatic necrosis syndrome (AHPNS) (13–17). The virulence factors which lead to pathogenicity in humans include thermostable direct hemolysin (TDH, encoded by the *tdh* gene), TDH-related hemolysin (encoded by the *trh* gene), and type 3 secretion systems 1 (T3SS1) and 2 (T3SS2) (18–21). Commonly, pathogenic V. parahaemolyticus is predominantly isolated from the stool samples of patients with gastroenteritis, whereas most strains isolated from environmental samples lack these virulence factors and are recognized as nonpathogenic strains (22).

Although birds are not a natural host of V. parahaemolyticus, aquatic birds feed on marine animals and several of those species are reservoirs of V. parahaemolyticus (23). Detection of *Vibrio* spp. in various types of birds has been reported in India (24), Japan (7), the USA (2, 25), Brazil (4), and Venezuela (6). A potentially pathogenic, *trh*-positive V. parahaemolyticus strain was previously isolated from ducks in Japan and from Manx shearwater (Puffinus puffinus) in Brazil (3, 7). Although the presence of V. parahaemolyticus in birds has been widely reported, genetic characterization and virulence gene profiling of this organism have not been performed. Multilocus sequence typing (MLST) is an efficient tool to achieve genetic characterization and study the molecular evolution of bacterial pathogens (26–29). An MLST analysis of V. parahaemolyticus strains isolated from aquatic birds in China led to the detection of isolates with identical sequence types (ST) from birds and marine animals inhabiting the same region, which supports the hypothesis that aquatic birds can acquire bacteria through the ingestion of prey animals (i.e., mollusks and fish), thus enabling bacterial transmission across geographical distances (1). In Thailand, the Bangpu Recreation Center, located in the Samut Prakan province, is recognized as a hot spot for migratory birds originating from various countries. The potential role of the birds in this area in the spread of pathogenic viruses was previously investigated (8); however, the information published to date does not cover bacteria. This study isolated and determined the genotypic profiles of V. parahaemolyticus from aquatic bird feces at the Bangpu Recreation Center using the MLST method. Five representative isolates possessing virulence genes were further selected for a comparative study of the T3SS2 region in bird- and human-pathogenic strains. The outcomes of this study generated new insights on the diversity and epidemiology of V. parahaemolyticus mediated by avian hosts.

## RESULTS

### Prevalence of *Vibrio parahaemolyticus* and virulence genes in aquatic bird fecal samples.

A total of 218 samples were collected over a period of 8 months at the Bangpu Recreation Center. The result of our analyses showed that 34.9% (76/218) of the samples were positive for V. parahaemolyticus based on both conventional culture methods and PCR of the species-specific gene *ldh.* A total of 308 *ldh*-positive isolates obtained from 76 samples were examined for the presence of virulence genes. Only 8 isolates from 5 samples (5/76; 6.6%) were positive for virulence genes. The hemolysin-encoding gene *trh* was the most frequently observed virulence gene (4/308; 1.3%), followed by *tdh* (3/308; 1%) and then by *tdh* and *trh* together (1/308; 0.3%). In addition, the distribution of T3SS-encoding genes was examined in all isolates. Testing for T3SS1 and T3SS2 in the 308 *ldh*-positive V. parahaemolyticus isolates showed that 100% (308/308) of them were positive for T3SS1 (encoded by *vopQ*), 1% (3/308) were positive for T3SS2α (encoded by *vopP*), and 1.6% (5/308) were positive for T3SS2β (encoded by *vopC*).

### Multilocus sequence typing of 49 *Vibrio parahaemolyticus* isolates from bird fecal samples.

Seven housekeeping genes were successfully amplified in the 49 isolates selected for MLST, and their nucleotide sequences were analyzed (Fig. S2). The 49 isolates represented 36 STs, of which 26 (72.2%) were novel (Table 1). The locus showing the highest nucleotide diversity was *dtdS* (31 nucleotides), followed by *pyrC* (29), *gyrB* (26), *recA* (25), *dnaE* and *pntA* (22 each), and *tnaA* (19). Novel alleles were assigned to each locus: *dtdS* (6), *dnaE* (3), *gyrB* (3), *pntA* (3), *pyrC* (3), *tnaA* (3), and *recA* (2). Of note, atypical *recA* genes of V. parahaemolyticus, which were previously described as resulting from interspecies horizontal gene transfer among bacteria in *Vibrionaceae*, were detected in five isolates, including MUVP22 (*recA*107), MUVP23 (*recA*107), MUVP24 (*recA*107), MUVP25 (*recA*276), and MUVP48 (*recA*276) (30).

**TABLE 1:** Allele profiles and sequence types of Vibrio parahaemolyticus isolates from aquatic bird fecal samples

No.	Isolate	Date of isolation (day-mo-yr)	Allele profile							ST
			Chromosome I genes			Chromosome II genes				
			*dnaE*	*gyrB*	*recA*	*dtdS*	*pntA*	*pyrC*	*tnaA*	
1	MUVP1	22/08/2016	28	39	230	19	253^*a*^	62	1	1922^*a*^
2	MUVP2	22/08/2016	158	23	153	74	66	154	33	1309
3	MUVP3	22/08/2016	341	51	98	444^*a*^	26	170	64	2011^*a*^
4	MUVP4	5/9/2016	158	23	153	74	66	154	33	1309
5	MUVP5	5/9/2016	158	23	153	74	66	154	33	1309
6	MUVP6	5/9/2016	158	23	153	74	66	154	33	1309
7	MUVP7	5/9/2016	158	23	153	74	66	154	33	1309
8	MUVP8	5/9/2016	158	507^*a*^	144	445^*a*^	254^*a*^	419^*a*^	266^*a*^	1923^*a*^
9	MUVP9	3/10/2016	28	106	82	251	18	38	2	985
10	MUVP10	17/10/2016	10	508^*a*^	15	446^*a*^	132	11	2	1924^*a*^
11	MUVP11	17/10/2016	5	106	59	78	50	328	17	1919
12	MUVP12	31/10/2016	234	285	74	278	61	78	57	1925^*a*^
13	MUVP13	31/10/2016	234	285	74	278	61	78	57	1925^*a*^
14	MUVP14	31/10/2016	234	285	74	278	61	78	57	1925^*a*^
15	MUVP15	31/10/2016	341	51	98	253	26	418^*a*^	24	1926^*a*^
16	MUVP16	30/01/2017	35	154	31	78	26	277	258	1927^*a*^
17	MUVP17	30/01/2017	35	154	31	78	26	277	258	1927^*a*^
18	MUVP18	14/02/2017	158	23	153	74	66	154	33	1309
19	MUVP19	14/02/2017	5	84	31	88	26	45	24	1928^*a*^
20	MUVP20	13/03/2017	248	506^*a*^	98	185	26	382	26	1929^*a*^
21	MUVP21	27/03/2017	3	82	62	180	30	7	267^*a*^	1930^*a*^
22	MUVP22	27/03/2017	11	48	107^*b*^	48	26	48	26	2516
23	MUVP23	27/03/2017	11	48	107^*b*^	48	26	48	26	2516
24	MUVP24	27/03/2017	11	48	107^*b*^	48	26	48	26	2516
25	MUVP25	27/03/2017	42	147	276^*b*^	136	66	296	214	2242^*a*^
26	MUVP26	14/11/2016	167	242	109	19	28	37	12	2229^*a*^
27	MUVP27	14/11/2016	11	106	192	220	71	73	17	1352
28	MUVP28	14/11/2016	36	285	292	13	49	227	24	2230^*a*^
29	MUVP29	14/11/2016	36	285	292	13	49	227	24	2230^*a*^
30	MUVP30	28/11/2016	3	25	60	144	31	128	26	288
31	MUVP31	28/11/2016	28	4	82	88	63	187	1	799
32	MUVP32	13/12/2016	31	221	395^*a*^	487^*a*^	26	45	23	2243^*a*^
33	MUVP33	13/12/2016	404^*a*^	187	31	488^*a*^	43	116	187	2244^*a*^
34	MUVP34	13/12/2016	403^*a*^	153	243	489^*a*^	272^*a*^	443^*a*^	26	2245^*a*^
35	MUVP35	13/12/2016	33	69	57	402	46	37	24	2231^*a*^
36	MUVP36	30/01/2017	377	147	67	206	23	37	280^*a*^	2246^*a*^
37	MUVP37	30/01/2017	35	154	31	78	26	277	258	1927^*a*^
38	MUVP38	30/01/2017	7	106	67	430	3	270	62	2233^*a*^
39	MUVP39	30/01/2017	291	129	25	39	18	3	20	2239^*a*^
40	MUVP40	14/02/2017	291	129	25	29	18	11	20	1879
41	MUVP41	27/02/2017	363	381	31	39	18	3	20	2240^*a*^
42	MUVP42	13/03/2017	28	177	140	390	45	257	54	2247^*a*^
43	MUVP43	13/03/2017	363	246	19	91	246	10	26	2241^*a*^
44	MUVP45	28/04/2017	402^*a*^	282	67	76	23	99	2	2248^*a*^
45	MUVP46	28/04/2017	28	106	82	204	18	7	26	491
46	MUVP47	28/04/2017	3	82	126	69	30	7	23	260
47	MUVP48	28/04/2017	42	147	276^*b*^	136	66	296	214	2242^*a*^
48	MUVP49	28/04/2017	167	58	396^*a*^	181	113	46	26	2249^*a*^
49	MUVP50	28/04/2017	167	58	396^*a*^	181	113	58	26	2250^*a*^

a Novel alleles or sequence types (STs).

b Atypical *recA* alleles of V. parahaemolyticus (30).

### Phylogenetic relationships among *Vibrio parahaemolyticus* isolates from bird fecal samples.

**FIG 1:**
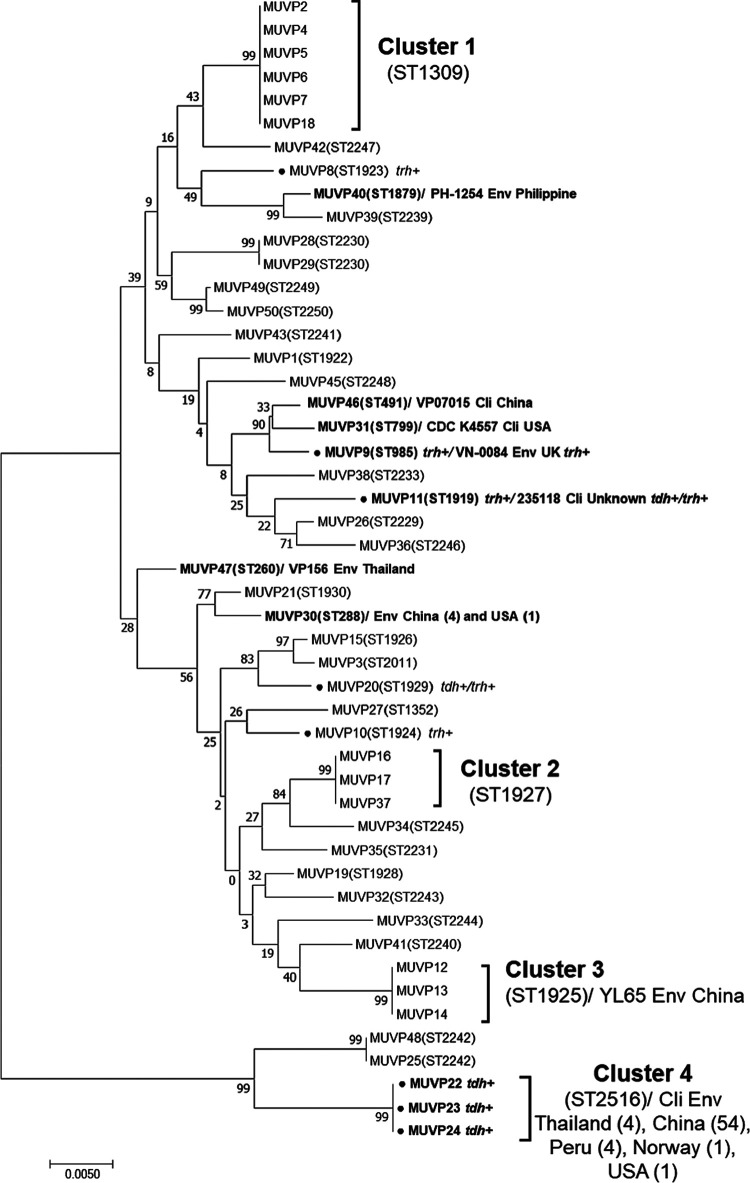
Phylogenetic tree of 49 V. parahaemolyticus isolates from aquatic bird fecal samples. The black circles in front of the names represent isolates possessing virulence genes. Bold letters represent isolates with sequence types (STs) identical to those available in the pubMLST database (https://pubmlst.org).

**FIG 2:**
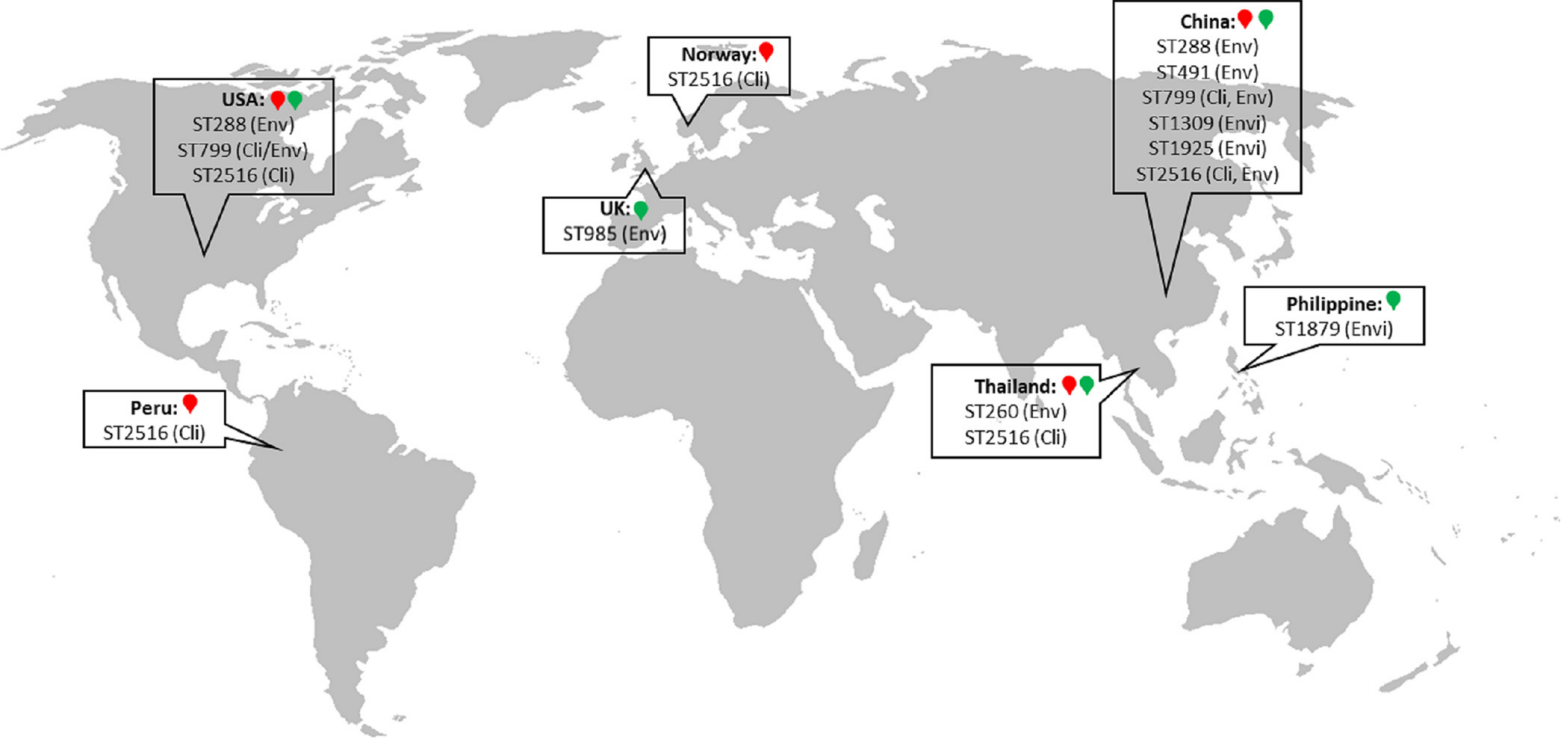
Geographical position of previously reported V. parahaemolyticus STs that were isolated from aquatic bird feces in Thailand (https://pubmlst.org).

A phylogenetic tree of the concatenated sequences of the seven housekeeping genes from the 49 V. parahaemolyticus isolates demonstrated that the isolates from bird feces had an overall diverse genetic background, with four distinct clusters, as follows: Cluster 1 (ST1309; *n* = 6), Cluster 2 (ST1927; *n* = 3), Cluster 3 (ST1925; *n* = 3), and Cluster 4 (ST2516; *n* = 3) (Fig. 1). Cluster 1 was composed of isolates with ST1309, which appeared to be the predominant ST detected in bird feces isolates. Of note, these ST1309 isolates were collected from different samples and at different time points. The MUVP2 isolate was obtained in August 2016, whereas MUVP4, 5, 6, and 7 were isolated a month later in September 2016, and MUVP18 was isolated in February of the following year (2017) (Fig. 1; Table 1). Clusters 2, 3, and 4 comprised isolates with ST1927, ST1925, and ST2516, respectively. In contrast to those in Cluster 1, the isolates within these clusters were from the same sample. However, several isolates collected from the same sample exhibited various STs, indicating the considerable genetic diversity of the V. parahaemolyticus population in individual sources (Table 1). The geographical positions of previously reported STs recovered from bird feces in Thailand demonstrated the global dissemination of pathogenic strains possibly carried by birds (Fig. 2). In particular, ST2516 was largely detected in clinical and environmental samples from the east and south coasts of China in Hangzhou, Shanghai, Zhejiang, and Guangdong from 2009 to 2016 (https://pubmlst.org/organisms/vibrio-parahaemolyticus) (accessed on 5 May 2022).

The large distant lineage of Cluster 4 was a consequence of atypical *recA* sequences (*recA*107). In Cluster 4, isolates MUVP22, MUVP23, and MUVP24 shared ST2516, which represents numerous clinical and environmental isolates which were previously reported in Thailand, China, Peru, Norway, and the USA (https://pubmlst.org/organisms/vibrio-parahaemolyticus) (accessed on 5 May 2022). Furthermore, three isolates from bird feces detected in this study represented STs which were identical to worldwide human clinical isolates, including MUVP46 (ST491; China), MUVP31 (ST799; USA), and MUVP11 (ST1919; unknown country) (Fig. 1). Seven isolates from bird feces represented STs which were identical to environmental isolates from various countries, including MUVP40 (ST1879; Philippines), MUVP9 (ST985; UK), MUVP47 (ST260; Thailand), MUVP30 (ST288; China), and MUVP12, 13, and 14 (ST1925; China).

Eight isolates possessing virulence genes were randomly distributed throughout the phylogenetic tree (Fig. 1). Among these isolates, MUVP8, MUVP10, and MUVP20 represented novel STs, whereas MUVP9, MUVP11, MUVP22, MUVP23, and MUVP24 shared STs with isolates previously reported in the pubMLST database. The MUVP9 isolate possessing *trh* represented ST985, which included the environmental isolate VN-0084, possessing *trh*, from the UK. Isolates MUVP22, MUVP23, and MUVP24 represented ST2516, which included clinical and environmental isolates in the pubMLST database; most of these isolates representing ST2516 possessed *tdh*. However, the MUVP11 isolate representing ST1919 contained only *trh*, whereas the clinical isolate 235118, from unknown sources in the pubMLST database, had both *tdh* and *trh*.

### Clonal relationships between the *Vibrio parahaemolyticus* isolates from aquatic bird fecal samples and strains from multiple sources in the pubMLST database.

**FIG 3:**
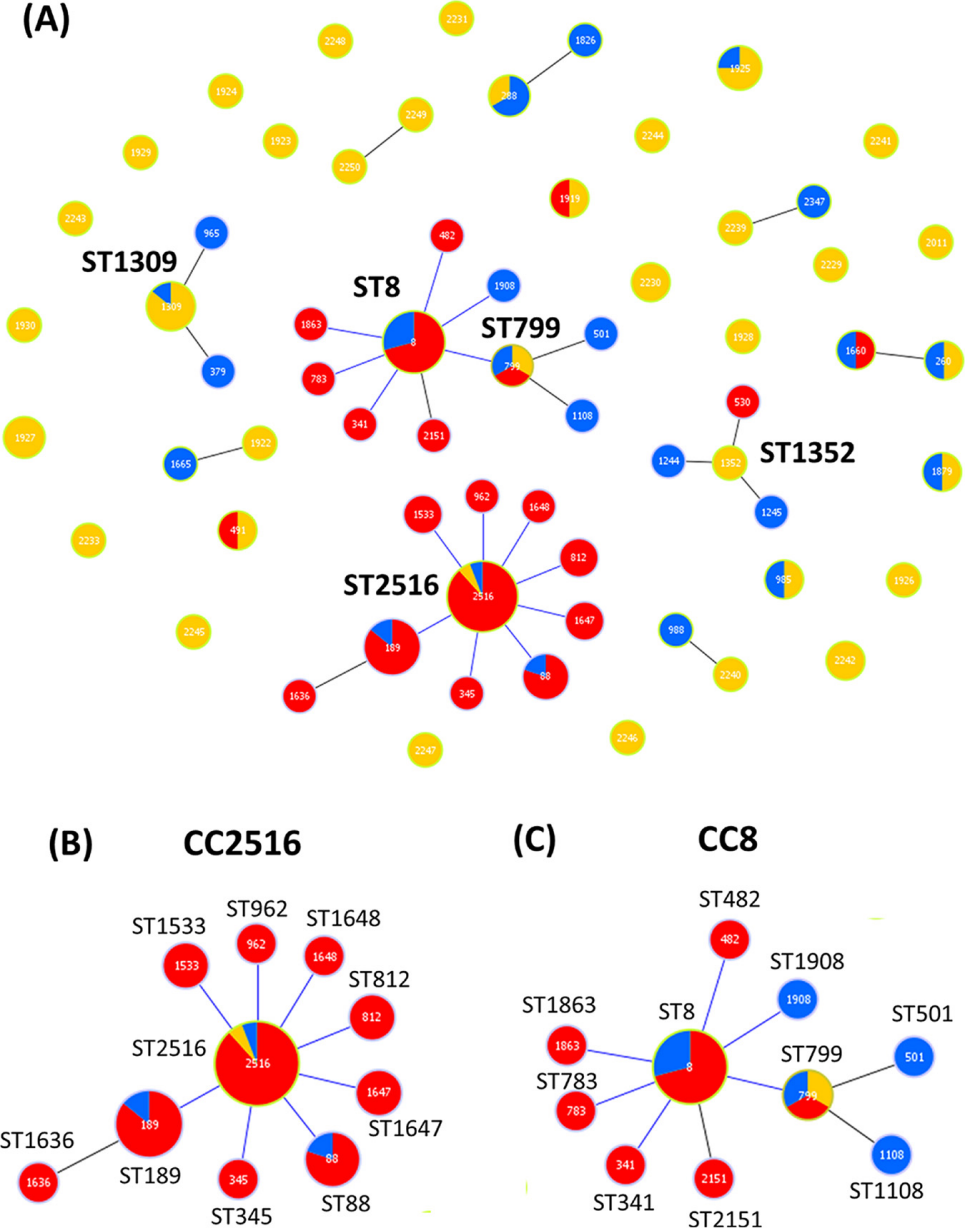
Population snapshot of V. parahaemolyticus isolates from bird feces and related STs from the pubMLST database. (A) eBURST diagram of all STs of the bird feces isolates and related STs from the pubMLST database. (B) Clonal complex (CC) 2516 with single-locus variants (SLVs). (C) CC8 with SLVs. Yellow color represents STs of bird feces isolates detected in this study. Red and blue colors represent STs of clinical and environmental isolates, respectively, from the pubMLST database.

A population snapshot of STs representing isolates from bird feces and related STs from the pubMLST database (https://pubmlst.org/vparahaemolyticus/) was illustrated using a goeBURST diagram (Fig. 3A). Two major clonal complexes (CCs), CC2516 and CC8, were identified as being associated with the isolates from bird feces. Isolates representing ST2516 appeared to be ancestral clones of CC2516 that showed eight single-locus variants (SLVs). ST2516 comprised 52 clinical isolates from China, Thailand, Peru, Norway, Bangladesh, India, Japan, and the USA (Fig. 3B and Table 2). The closely related ST189 represented pathogenic strains that had been previously detected (more than 2 decades ago) in Asia (1984 to 1999) and were subsequently found in other parts of the world, including the USA in 2007. Furthermore, ST88 was previously identified (before 1995) as the predominant clone responsible for V. parahaemolyticus infections in Peru (31). The MUVP31 (ST799) isolate from bird feces was a part of CC8, which has been well recognized as the pathogenic clone causing an outbreak in Asian-Pacific countries, as well as in specific areas of the USA and Canada (Fig. 3C; Table 2). In addition, CC1352 (ST1352) and CC1309 (ST1309) of bird isolates were founders of minor CCs (Fig. 3A). ST1352 was closely related with clinical isolate ST530 from China, whereas ST1309 was linked exclusively with environmental isolates from Thailand and China (Fig. 3A; Table 2).

**TABLE 2:** Sequence types in clonal complexes and single-locus variants closely related to V. parahaemolyticus isolates from aquatic bird feces (https://pubmlst.org/)^*a*^

CC or doublet	STs	Frequency (no. of strains)^*b*^	Country (no. of strains)	Yr of isolation	Source(s) (no. of strains)^*c*^
2516	2516	64	Thailand (4), China (54), Peru (4), Norway (1), USA (1)	1990–2018	B (3), C (58), E (3)
	189	30	Thailand (1), China (24), India (2), Japan (2), USA (1)	1984–2017	C (27), E (3)
	88	5	Peru (3), USA (1), Bangladesh (1)	1982–1997	C (4), E (1)
	345	1	China	2010	C
	812	2	China	2008	C
	962	1	China	2008	C
	1533	1	China	2014	C
	1647	2	China	2014	C
	1648	1	China	2014	C
799	799	4	Thailand (1), USA (2), China (2)	2006–2021	B (1), C (1), E (3)
	8	38	China (19), Japan (2), Philippines (1), India (1), Thailand (1), USA (12), Canada (2)	1984–2021	C (30), E (8)
	501	1	China	2008	E
	1108	1	China	2006	E
1352	1352	1	Thailand	2016	B
	530	1	China	2006	C
	1244	1	China	2013	E
	1245	1	China	2013	E
1309	1309	8	Thailand (6), China (2)	2009–2017, 2009	B (6), E (2)
	379	1	China	2005	E
	965	1	China	2010	E
260	260	2	Thailand	2017, 2003	B (1), E (1)
	1660	2	China	2014	C (1), E (1)
288	288	6	Thailand (1), China (4), USA (1)	2007–2021	B (1), E (5)
	1826	1	China	2014	E
1922	1922	1	Thailand	2016	B
	1,665	1	China	2014	E
2239	2239	1	Thailand	2017	B
	2347	1	China	2016	E
2240	2240	1	Thailand	2017	B
	988	1	China	2006	E
2249	2249	1	Thailand	2017	B
	2250	1	Thailand	2017	B

a CC, clonal complex; ST, sequence type.

b Number of strains in the V. parahaemolyticus MLST database, including the isolates identified in this study.

c C, clinical; E, environmental; B, bird feces; NA, information not available.

An eBURST analysis identified six doublets, including ST288, ST1922, ST2239, ST2240, ST260, and ST2249 (Fig. 3A, Table 2). ST288, ST1922, ST2239, and ST2240 were linked with STs containing environmental isolates from China. ST260 was the only doublet that was linked with the Chinese clinical ST1660. Lastly, ST2249 was linked with ST2250, an isolate from bird feces which was identified in this study. The remaining 25 STs were individual, unlinked STs, so-called singletons. Although most of these singletons were novel STs, five (ST491, ST1919, ST985, ST1879, and ST1925) were identical with STs of clinical and environmental isolates from China, the Philippines, the UK, and the USA (Fig. 1).

### Presence of a pathogenicity island harboring type three secretion system 2 in *Vibrio parahaemolyticus* isolated from bird feces.

T3SS2 is a well-known virulence factor encoded by a gene located in the VPaI-7 pathogenicity island on chromosome 2 of V. parahaemolyticus (32). T3SS2 is divided into two types, T3SS2α and T3SS2β, with T3SS2α being related to *tdh+/trh−* isolates and T3SS2β being related to *trh+/tdh+* or *trh+* isolates (33). Whole-genome sequencing (WGS) of the five selected isolates from bird feces possessing virulence genes (MUVP8, MUVP9, MUVP10, MUVP20, and MUVP22) were analyzed for the presence of virulence genes. The distributions of the T3SS2α- and T3SS2β-encoding genes of V. parahaemolyticus isolated from bird feces in this study are presented in Tables 3 and 4, respectively. For T3SS2α-related gene analysis, V. parahaemolyticus RIMD2210633 (*tdh+/trh−*) was used as the reference strain. We found that MUVP22 contained T3SS2α-related genes identical to those of the RIMD2210633 strain (Table 3). Those genes encoded apparatus proteins (*vscS2*, *vscN2*, *vscC2*, *vscT2*, *vscR2*, *vscU2*, and *vcrD2*), translocons (*vopD2* and *vopB2*), and effectors (*vopC*, *vopL*, and *vopP*). The MUVP10 and MUVP20 isolates did not share T3SS2α genes with the reference strain RIMD2210633, with the exception of *vscR2*. For T3SS2β-related gene analysis, V. parahaemolyticus TH3996 (*tdh−/trh+*) was used for the reference strain. We observed that MUVP8, MUVP9, MUVP10, and MUVP20 possessed identical T3SS2β-related genes compared with the reference strain, including genes encoding apparatus proteins (*vscS2*, *vscN2*, *vscC2*, *vscT2*, *vscR2*, *vscU2*, and *vcrD2*), translocons (*vopD2* and *vopB2*), and effectors (*vopC*, *vopL*, and *vopP*). Among all T3SS2β-related genes, only *vscR2* was detected in the MUVP22 isolate. The gene organization of the T3SS2α- and T3SS2β-related gene cassette of bird V. parahaemolyticus was highly similar to those of the reference strains RIMD2210633 and TH3996, respectively (Fig. S3 and S4 in the supplemental material). Nevertheless, several insertions and deletions of hypothetical protein-coding genes were observed in V. parahaemolyticus isolates from birds.

**TABLE 3:** Distribution of T3SS2α-related genes in V. parahaemolyticus strains

Strain	Hemolysin gene		T3SS2α-related genes											
			Apparatus							Translocon		Effector		
	*tdh*	*trh*	*vscS2*	*vscN2*	*vscC2*	*vscT2*	*vscR2*	*vscU2*	*vcrD2*	*vopD2*	*vopB2*	*vopC*	*vopL*	*vopP*
RIMD 2210633	+	−	+	+	+	+	+	+	+	+	+	+	+	+
MUVP8	−	+	−	−	−	−	−	−	−	−	−	−	−	−
MUVP9	−	+	−	−	−	−	−	−	−	−	−	−	−	−
MUVP10	−	+	−	−	−	−	+	−	−	−	−	−	−	−
MUVP20	+	+	−	−	−	−	+	−	−	−	−	−	−	−
MUVP22	+	−	+	+	+	+	+	+	+	+	+	+	+	+

**TABLE 4:** Distribution of T3SS2β-related genes in V. parahaemolyticus strains

Strain	Hemolysin gene		T3SS2β-related genes											
			Apparatus							Translocon		Effector		
	*tdh*	*trh*	*vscS2*	*vscN2*	*vscC2*	*vscT2*	*vscR2*	*vscU2*	*vcrD2*	*vopD2*	*vopB2*	*vopC*	*vopL*	*vopP*
TH3996	−	+	+	+	+	+	+	+	+	+	+	+	+	+
MUVP8	−	+	+	+	+	+	+	+	+	+	+	+	+	+
MUVP9	−	+	+	+	+	+	+	+	+	+	+	+	+	+
MUVP10	−	+	+	+	+	+	+	+	+	+	+	+	+	+
MUVP20	+	+	+	+	+	+	+	+	+	+	+	+	+	+
MUVP22	+	−	−	−	−	−	+	−	−	−	−	−	−	−

### Comparative phylogenetic tree of whole-genome and MLST sequences of *Vibrio parahaemolyticus* from bird feces and those from multiple worldwide sources in GenBank.

**FIG 4:**
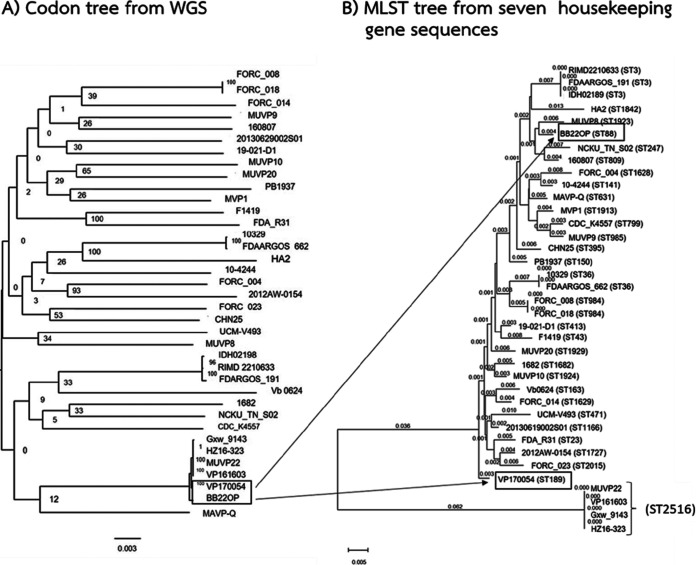
Phylogenetic tree of 32 V. parahaemolyticus strains from GenBank and five isolates from bird fecal samples. (A) The codon tree was constructed using whole-genome sequencing data by an algorithm installed in PATRIC (34). (B) The multilocus sequence typing tree was constructed based on seven housekeeping gene sequences using MEGA 7.0 (80).

The WGS data of 32 V. parahaemolyticus strains, including strains from clinical sources (*n* = 16) and environmental sources (*n* = 16) with geographically diverse origins, were obtained from GenBank and incorporated with the WGS results of our five bird V. parahaemolyticus isolates to construct a phylogenetic tree using a shared homolog amino acid cluster algorithm, codon tree (34, 35). A comparison of the codon tree and MLST tree for a total of 37 isolates was performed. Although the topology of the two trees was similar, we observed an incongruent relationship of strains sharing ST2516 (Fig. 4). The codon tree represented a tight cluster of isolate MUVP22 and clinical strains from China (Gxw_9143, HZ16-323, VP161603, and VP170054) and an environmental strain from Bangladesh (BB22OP) (Fig. 4A). However, strains VP170054 (ST189) and BB22OP (ST88) were located distantly from each other, and from MUVP22, in the MLST tree (Fig. 4B). These results reflect the pitfalls of MLST analysis because the horizontal gene transfer of a housekeeping gene (*recA*) caused an inaccurate evolution of the phylogenetic tree. Nevertheless, the results from codon tree supported the information from goeBURST (Fig. 3), which showed that V. parahaemolyticus from the bird feces isolate MUVP22 was involved in the lineage of pathogenic V. parahaemolyticus. In particular, the BB22OP strain, a pre-pandemic strain isolated from the environment in Bangladesh in the early 1980s, appeared closely to MUVP22 (Fig. 4A). Together with the goeBURST diagram (Fig. 3), these findings strongly indicated that aquatic birds can carry strains that are closely related to pre-pandemic clones with subsequent genetic changes.

## DISCUSSION

This research clearly showed that V. parahaemolyticus isolates from aquatic bird feces had a tremendously diverse genetic background. The majority of the isolates represented novel STs (72.2%), and isolates with multiple STs were recovered from a single bird fecal sample. Commonly, the gut of humans and animals provides a reservoir which facilitates horizontal gene transfer among the resident microbiota, contributing to an extremely high microbial diversity (36). Other factors, including a stable temperature, host diet, and an extremely high density of microbial cells, have enabled the gut to become the most favorable ecological niche for horizontal gene exchange (37). In Escherichia coli, bacteriophage-mediated horizontal gene transfer plays a vital role in the evolutionary selection that leads to the emergence of new commensal strains in the mouse gut (38). A previous study showed that asymptomatic humans can be reservoirs of V. parahaemolyticus with various serotypically different strains (39). In this study, the genetically diverse V. parahaemolyticus isolates identified in bird feces possibly occurred as a consequence of the biological and physiochemical conditions of the avian gut, which may enhance horizontal gene transfer among inhabiting bacteria and contribute to the emergence of new strains.

Among the four distinct clusters identified in the MLST phylogenetic tree, the largely distinct lineage of Cluster 4 (MUVP22, MUVP23, and MUVP24) and isolates MUVP25 and MUVP28 was a consequence of interspecies recombination in *recA* (30). Bird isolates in Cluster 4 (ST2516) and most worldwide isolates representing ST2516 also shared a common serotype, O4:K8. ST2516 was initially reported as ST265 in previous publications (30, 40–42) before subsequent reclassification as ST2516 in the pubMLST database (accessed 5 May 2022). ST2516 corresponds to widespread clinical isolates from Thailand (in year 1990), China (from year 1990 to 2019), Peru (in year 1996 and 2007), Norway (in year 2018), and USA (in year 2007) (Table 2). In Peru, the ST2516 variant replaced the previous predominant pathogenic clone, ST88, in 1995 (42). Subsequently, ST2516 and its variants were replaced by the Asian pandemic clone, ST3, in 1997 (31). However, it is known that the genetic diversity of V. parahaemolyticus is likely driven by homologous recombination (43–45). Among 1,274 public V. parahaemolyticus genomes, 84 genomes (6.6%) harbor interspecies recombined *recA* suggesting that such event is commonly occurred in V. parahaemolyticus (46). The interspecies recombination in *recA* affected the topology of the MLST phylogenic tree, which obscured the true evolution of this organism. The eBURST algorithm, which was subsequently developed to the goeBURST algorithm (47), has been proposed as a suitable tool to investigate the ascent of pandemic clones and the population structure of bacterial pathogens with a frequent genetic recombination background (22, 48–51). In the present study, the population snapshot provided by goeBURST demonstrated the clonal relationships between the bird isolates and global related strains in the wider perspective compared with the MLST phylogenetic construction. In the goeBURST diagram, two major CCs, CC2516 and CC8, were identified (Fig. 3). CC2516 comprised eight SLVs, including ST189, ST88, ST962, ST33, ST647, ST648, ST812, and ST345. In particular, ST189 was previously reported as a widely detected clinical clone in Asia during the period of 1984 to 2017 (Table 2). Furthermore, ST88 included a clinical pre-pandemic clone that was originally recovered in Bangladesh and subsequently transmitted to Peru and had been circulated in the country until 1997 (30, 31, 42). Moreover, ST88 was identified in pandemic isolates in Zhejiang, a coastal province in China, during the short period spanning the years from 2010 to 2012 (52). Other STs representing an SLV with ST2516 were clinical isolates recovered from China. In addition, the goeBURST diagram demonstrated an SLV connection between the aquatic bird isolate ST799 and the worldwide ST8, which was a founder of CC8 (Fig. 3). ST8 was responsible for the outbreak of V. parahaemolyticus infection by raw oyster consumption reported in MD, USA, in 2010 (41). Before the ST8 clone was identified in Maryland, it had been widely isolated from clinical samples, particularly in Asian countries, including China (1994 and 2008), India (1999), Japan (1984), the Philippines (1998), and Thailand (2006); and in Canada in 2006 and 2007 (https://pubmlst.org/organisms/vibrio-parahaemolyticus). It was speculated that the transmission of ST8 strains from Asia to the eastern coast of the USA probably occurred via the ballast water transported by commercial ships coming from Asia, ocean currents, and events introducing exotic fish from non-native strains into that area (41). Obviously, the isolates within CC2516 are more likely to have pathogenic potential than other identified CCs because CC2516 contains a large number of clinical isolates reported globally and they have an almost identical pattern of virulence gene cassette T3SSα with the pandemic clone O3:K6 (RIMD2210633). However, the pathogenic potential of isolates within CC8 are not neglectable since they were evidenced for intercontinental transmission (41). The evidence from our study strongly suggests that aquatic birds are potential carriers of V. parahaemolyticus and possibly participate in the transmission of this organism across large geographical distances (Fig. 2).

Satellite tracking of brown-headed gulls (*C. brunnicephalus*) in Bangpu (Samut Prakan, Thailand) showed the flyway of migratory birds in this area from their breeding ground in China to Southeast Asian countries, including Bangladesh, India, Myanmar, Thailand, Cambodia, and Vietnam (8). V. parahaemolyticus isolates from bird feces detected at Bangpu represented identical STs to those of environmental and clinical strains from China (ST288, ST491, ST799, ST1309, ST1925, and ST2516) (Fig. 1 and 2). The clustering analysis also supported the close relationships between isolates from bird feces collected at Bangpu and strains from China (Fig. 3; Table 2). It is conceivable that aquatic birds could take up local V. parahaemolyticus via the ingestion of sea animals, seaweed, and plankton along the coastal area and transmit the organism to the territory they visit. In addition, evidence that birds acquired *Vibrio* spp. through the direct predation of local mollusks was provided by a study conducted at the Liaohe River, China, which reported a total of 19 V. parahaemolyticus isolates with eight STs, including three novel STs (37.5%), from aquatic birds (1). Our study reported a total of 49 V. parahaemolyticus isolates from aquatic birds representing 36 STs, 26 of which were novel (72.2%). Comparatively, the V. parahaemolyticus isolates obtained from birds at Bangpu were more diverse than the strains from the Liaohe River. This result indicates the extremely high genetic variation of V. parahaemolyticus isolates in Thailand, even though the sampling area at Bangpu was much more restricted than that at the Liaohe River, which covered three sampling sites, including Yingkou, Panjin, and Shanghai. In addition, sampling time points and the number of isolates may affect the level of diversity. The Liaohe isolates were obtained from fecal samples collected at three time points during October 2017 and March 2018, whereas the isolates from Bangpu were obtained from samples collected at 17 time points from August 2016 to April 2017.

This study showed that strains isolated from bird feces possessed *tdh* (*n* = 3), *trh* (*n* = 4), or both *tdh* and *trh* (*n* = 1). In general, a higher proportion of *trh*-positive versus *tdh*-positive V. parahaemolyticus was detected among environmental samples (53–55). In Japan, *trh*-positive V. parahaemolyticus strains with various serotypes have been isolated from aquatic birds (7). However, our results revealed a similar distribution of *tdh*- and *trh*-positive V. parahaemolyticus isolated from bird feces (Table 1). Moreover, unpublished data from our group showed that the detection rate of pathogenic V. parahaemolyticus possessing *tdh* and/or *trh* in aquatic bird feces at Bangpu (2.6%; *n* = 308) was higher than that of isolates obtained from seawater in the same area (1.9%; *n* = 623). It is possible that the avian gut provides more favorable conditions for the survival of pathogenic strains than the natural environment. Our study identified T3SS2α and T3SS2β in V. parahaemolyticus isolates from aquatic bird feces, similar to previous studies which reported T3SS2α and T3SS2 in isolates from environmental samples, including seafood, sediment, and seawater (56–61). Nevertheless, our analysis also demonstrated the highly similar gene organization of isolates from aquatic bird feces compared with that of the reference clinical isolates RIMD2210633 and TH3996 (Tables 3 and 4) (Fig. S3 and S4 in the supplemental material) (20, 33), strongly supporting the virulence potential of V. parahaemolyticus isolates from aquatic birds. The list of identified T3SS2 genes in aquatic bird isolates was also consistent with previous literature describing a pathogenicity island harboring T3SS2 in V. parahaemolyticus (62–64).

A comparative study of the core genome MLST (cgMLST) (2,254 core genes) and conventional MLST (seven housekeeping genes) suggested that cgMLST can delineate subpopulations of V. parahaemolyticus strains within the same ST into distinguishable groups based on epidemiological data, including outbreak, serovariant, and geographical origin (65). V. parahaemolyticus inhabits a wide range of marine environments, with multiple life stages as a free-living organism in seawater, a host-associated organism in sea animals, and a pathogen in the human gut. Thus, variations in lifestyle result in its extremely diverse nature genomic background, with the capability to adapt and survive under various coastal conditions (22, 65). In addition, the genetic background of this organism has been affected by homologous recombination and horizontal gene transfer, which were necessary for rapid adaptation to environmental changes (30, 42, 43, 66, 67). These genetic features underlie the non-robust MLST interpretation. Our results supported those of previous studies which showed that WGS analyses could yield enhanced resolution for V. parahaemolyticus classification; nevertheless, conventional MLST is a reliable tool that can generate phylogenetic data for the V. parahaemolyticus genome when WGS is unavailable (68). The limitation in our study was the uncertain origin of bird fecal samples. Although most of the aquatic birds inhabiting Bangpu during the sampling period were migratory birds, it was plausible that a small number of these had previously adapted to this area and become sedentary at the time of collection. Thus, it was unlikely that the obtained V. parahaemolyticus isolates in this study were solely from migratory birds. In summary, this research successfully isolated and performed genetic characterization of V. parahaemolyticus from aquatic bird feces in Thailand. Our findings provide clear evidence that aquatic birds harbor potentially pathogenic V. parahaemolyticus, indicating their role in the dissemination and epidemics of V. parahaemolyticus in coastal areas.

## MATERIALS AND METHODS

### Aquatic bird fecal sample collection.

Fecal samples from aquatic birds (dominant species *Chroicocephalus brunnicephalus*) at Bangpu, (Samut Prakan, Thailand) were collected twice a month from August 2016 to March 2017 (Fig. S1 in the supplemental material). The selected fecal samples had been observed to be recently dropped from aquatic birds to ensure the intact condition of the samples. All selected samples appeared highly moist and located distantly from other feces. Approximately 1 g of each fecal sample was collected using a sterile cotton swab which was previously soaked in normal saline solution and swabbed into the fecal samples on the ground, followed by direct streaking on a selective medium plate, i.e., thiosulfate citrate bile salt agar (TCBS, Difco, Detroit, MI). The swab was then placed in 10 mL of alkaline peptone water (APW, Difco) containing 3% (wt/vol) sodium chloride (NaCl, Merck, Darmstadt, Germany) at each sampling site. Both the TCBS agar plates and the enrichment broth of APW containing 3% (wt/vol) NaCl were incubated at 37°C for 18 to 24 h within 5 h after transfer to the laboratory.

### Isolation and identification of *V. parahaemolyticus* strains.

After the incubation described above, bacterial cultures from enrichment APW with 3% (wt/vol) NaCl were subcultured on TCBS agar at 37°C for 18 to 24 h. Subsequently, one to three suspected green colonies of V. parahaemolyticus from direct TCBS plates and TCBS plates enriched by APW with 3% (wt/vol) NaCl were randomly selected. In total, three to five colonies were collected for each sample. The colonies obtained were biochemically characterized according to a published method (69). The identified isolates were then confirmed for species-specific *ldh* of V. parahaemolyticus by PCR (70). Pure cultures were preserved using 20% (vol/vol) glycerol in Luria-Bertani (LB, Becton Dickinson, Franklin Lakes, NJ) broth containing 3% (wt/vol) NaCl and stored at −80°C for further analysis.

### Detection of virulence genes.

Chromosomal DNA was prepared according to the methods of a previous study (16). The presence of virulence genes, including *tdh*, *trh*, *vopQ* (encoding T3SS1), *vopP* (encoding T3SS2α), and *vopC* (encoding T3SS2β), was determined using published PCR primers (33, 70–73) (Table S1 in the supplemental material). The PCR conditions used here were adapted from a previous method (16). Finally, the PCR products were analyzed by 2% (wt/vol) agarose gel (Sigma-Aldrich, St. Louis, MO) electrophoresis.

### Multilocus sequence typing analysis.

A total of 49 V. parahaemolyticus isolates were selected for MLST analysis by considering the virulence gene profiles and the distributed times of collection throughout the sampling period (Table 1). We found that eight of the 49 isolates possessed virulence genes, including *tdh* and/or *trh*, *vopQ* (T3SS1), *vopP* (T3SS2α), and *vopC* (T3SS2β) (Table S2).

PCR amplification and nucleotide sequencing of seven housekeeping genes were performed as described previously (74). The amplified PCR products were purified using the QIAquick purification kit (Qiagen, Hilden, Germany) according to the manufacturer’s instructions. DNA sequencing was performed on an ABI 3730XL platform (Applied Biosystems, Carlsbad, CA) using the BigDye v3.1 Cycle Sequencing Kit (Applied Biosystems). The obtained V. parahaemolyticus nucleotide sequences were analyzed using Geneious version 11.0.5 (75). The allele number and ST of each V. parahaemolyticus strain were determined by comparison with the pubMLST V. parahaemolyticus database (http://pubmlst.org/vparahaemolyticus/) (43).

### Whole-genome sequencing.

Whole-genome sequencing of the representative five *V. paraheamolyticus* isolates possessing virulence genes was performed at the Omics Sciences and Bioinformatics Center (Chulalongkorn University, Bangkok, Thailand) according to the institute’s protocol. Briefly, 100 ng of genomic DNA was subjected to DNA sequencing library preparation using the Qiagen QIAseq FX DNA Library kit (Qiagen). Genomic DNA was fragmented using an enzymatic reaction and cleaned with magnetic beads for library preparation. An adaptor index was ligated to the fragmented DNA. The quality and quantity of the indexed libraries was measured using an Agilent 2100 Bioanalyzer and a DeNovix fluorometer. Cluster generation and paired-end 2 × 250 nucleotide read sequencing were performed on an Illumina MiSeq sequencer.

### Phylogenetic analysis.

A nucleotide sequence alignment of concatenated housekeeping genes (3,669 bp) was performed using ClustalW (76). The evolutionary history was inferred using the neighbor-joining method (77). The percentage of replicate trees in which the associated taxa clustered together in the bootstrap test (500 replicates) was determined as described previously (78). Evolutionary distances were computed using the Jukes-Cantor method (79). Evolutionary analyses were conducted in MEGA7 (80).

### Genome sequence analysis.

The quality check of raw sequence reads and genome assembly was performed using the platform of the Pathosystems Resource Integration Center (PATRIC) (34). Sequence trimming was performed by Trim Galore (81). Quality control of trimmed sequences was carried out by FastQC (82). Alignment of the tested genomes with the reference genome (V. parahaemolyticus RIMD2210633) was conducted using Bowties (83, 84). Genome annotation was performed using RASTtk (85). WGS of five V. parahaemolyticus in this study and other worldwide isolates obtained from GenBank were used to construct a codon tree by an algorithm installed in PATRIC (https://patricbrc.org/app/PhylogeneticTree).

### *In silico* detection and comparative nucleotide analysis of virulence genes.

Virulence factor prediction was performed at PATRIC using the Virulence Factor Database (VFDB) (86). The pathogenicity islands of all five strains from bird feces were identified by BLAST option using the T3SS2 sequence of strain RIMD2210633 as a reference for T3SS2α and the T3SS2 sequence of strain TH3996 as a reference for T3SS2β, with the maximum hit set at 50 and the E value set at 10. After obtaining the hit sequence files, we visualized the comparative gene organization using the Artemis Comparison Tool (ACT) (87).

### Data availability.

The genome sequences of V. parahaemolyticus isolates MUVP8, MUVP9, MUVP10, MUVP20, and MUVP22 were deposited in the GenBank/DDBJ/EMBL databases under accession no. JALAZC000000000, JALAZB000000000, JALAZA000000000, JALAYZ000000000, and JALAYY000000000, respectively.
