# Supplementary material for: Multilocus Sequence Typing and Virulence Potential of Vibrio parahaemolyticus Strains Isolated from Aquatic Bird Feces
Source: Microbiol Spectr. 2022 Jun 13;10(3):e00886-22. doi: 10.1128/spectrum.00886-22 (PMC9241773; doi:10.1128/spectrum.00886-22)
Supplement: Supplemental file 1 — Supplemental material. Download spectrum.00886-22-s0001.pdf, PDF file, 0.4 MB [file spectrum.00886-22-s0001.pdf]

Supplemental materials

Figure S1

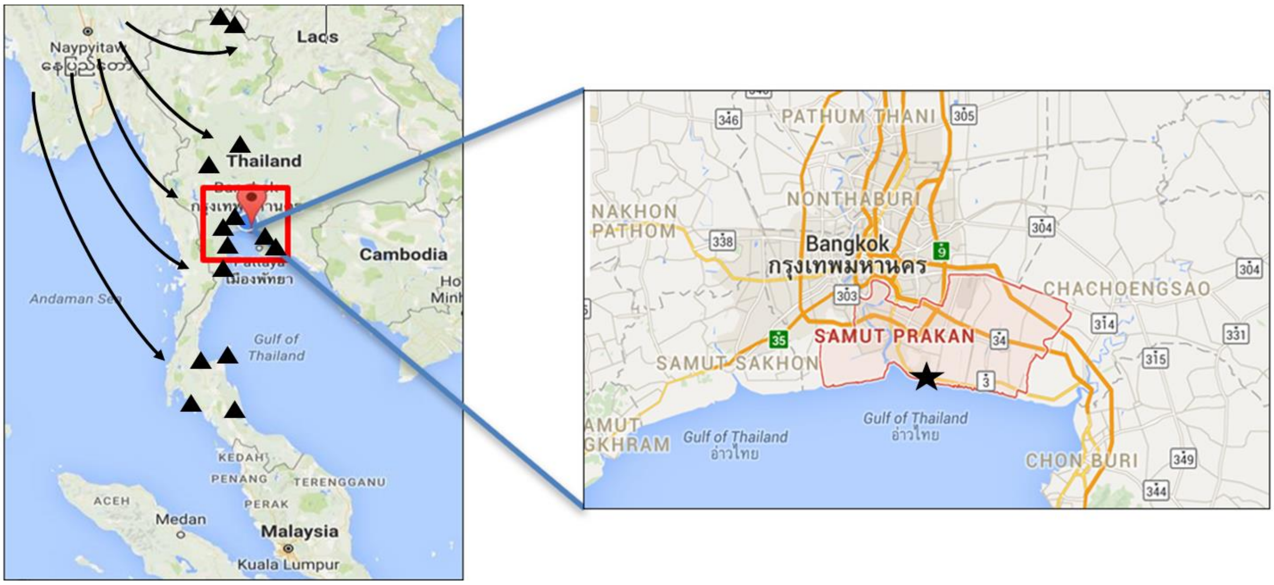

Figure S1 Map of sampling site at Bangpu Recreational Center (black star), Samut Prakan, Thailand. Arrows represent migratory direction of Brown-headed gull (*Chroicocephalus brunnicephalus*) from central Asia to inland lakes and coastal regions in southern Asia including Thailand. Triangles represent range map of *C. brunnicephalus* in Thailand. Data provided by Thai National Parks (2022).

Figure S2

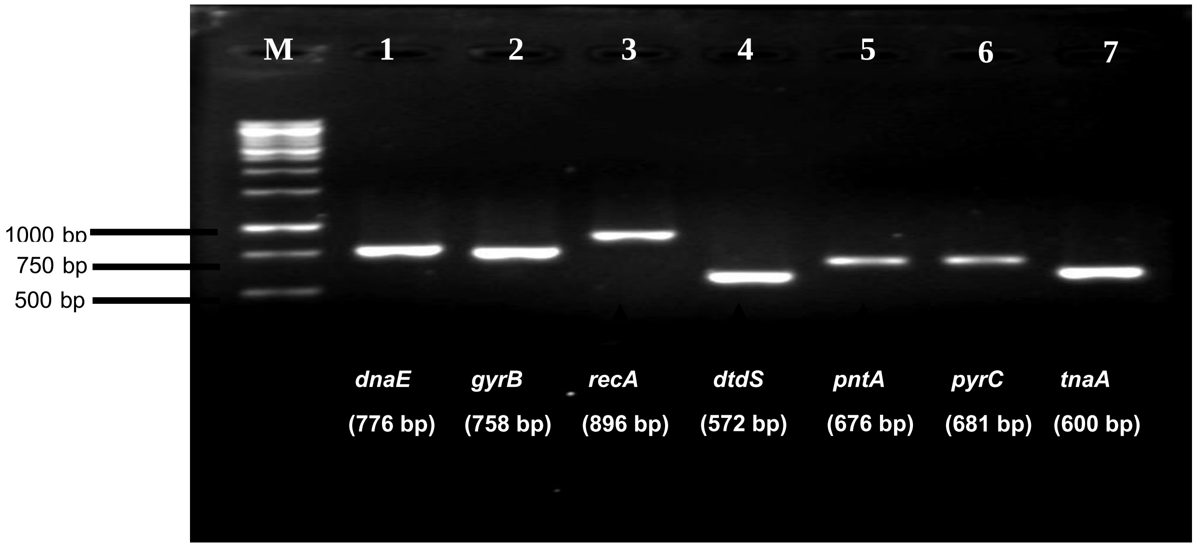

Figure S2 The 1% (w/v) agarose gel electrophoresis of *V. parahaemolyticus* PCR products of 7 housekeeping genes positive control of strain VP902. Lane M: 1 kb ladder (Thermo Scientific, USA); Lane 1-7: PCR products of 7 housekeeping genes including *dnaE* (776 bp), *gyrB* (758 bp), *recA* (896 bp), *dtdS* (572 bp), *pntA* (676 bp), *pyrC* (681 bp) and *tnaA* (600 bp), respectively

Figure S3

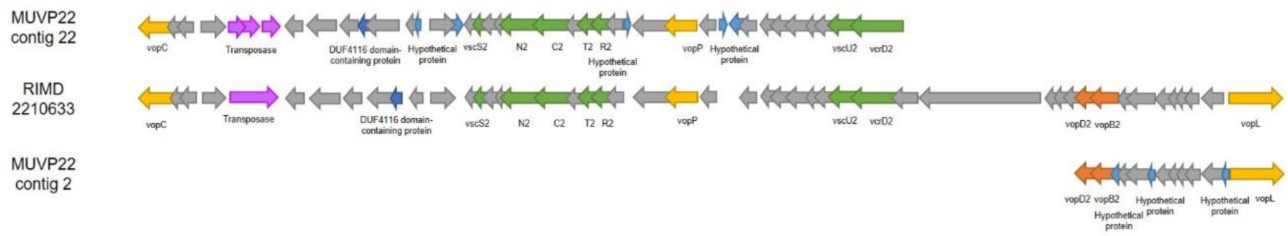

Figure S3 Comparison of the gene organization of T3SSα-related genes in *Vibrio parahaemolyticus* RIMD2210633 and MUVP22. Green arrows indicate the gene encoding putative apparatus protein of T3SS2α, yellow arrows indicate the gene encoding putative effector protein of T3SS2α, orange arrows indicate the gene encoding putative translocon protein of T3SS2α, purple arrows indicate transposase genes, dark blue arrows indicate rearranged DUF4116 domain containing protein, light blue arrows indicated additional hypothetical protein, and gray arrows indicate other proteins.

Figure S4

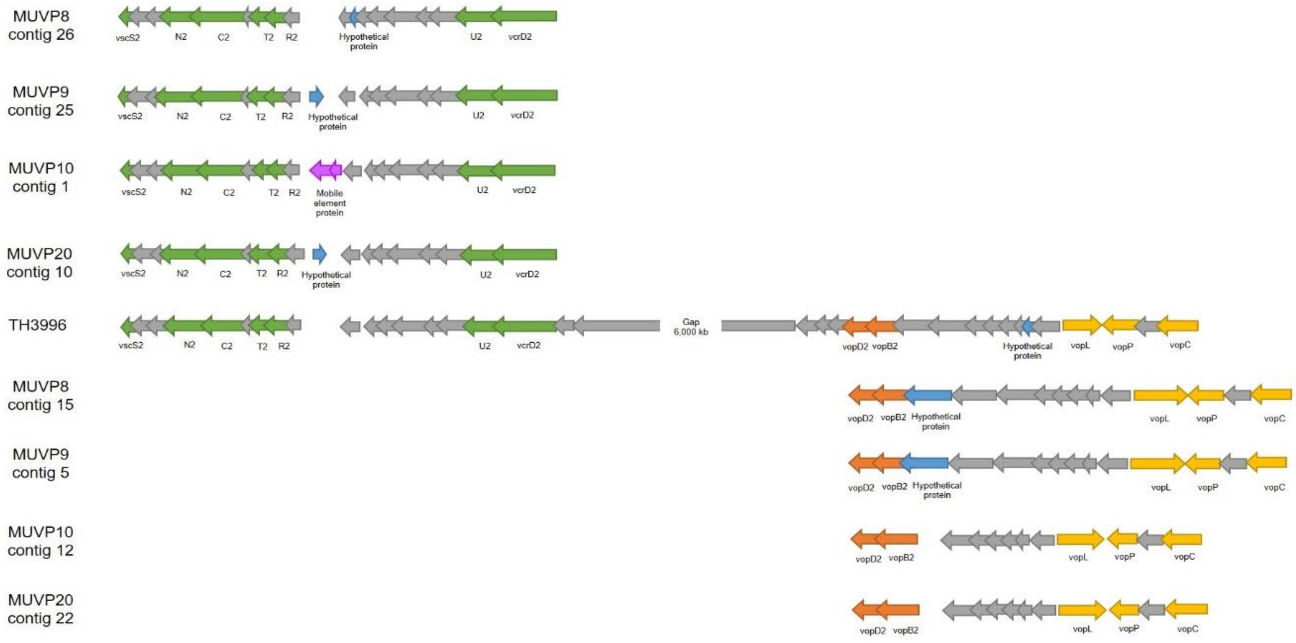

Figure S4 Comparison of the gene organization of T3SS2β-related genes in *Vibrio parahaemolyticus* TH3996, MUVP8, MUVP9, MUVP10 and MUVP20. Green arrows indicate the gene encoding putative apparatus protein of T3SS2β, yellow arrows indicate the gene encoding putative effector protein of T3SS2β, orange arrows indicate the gene encoding putative translocon protein of T3SS2β, purple arrows indicate mobile element protein, light blue arrows indicated additional hypothetical protein, and gray arrows indicate other proteins.

Table S1 Nucleotide sequence of primers used for detection of species specific and virulence genes of *Vibrio parahaemolyticus*

Gene	Sequence (5' to 3')	Product size (bp)	Reference
ldh[a]	Forward5'AAAGCGGATTATGCAGAAGCACTG3'<br>Reverse 5'-GCTACTTTCTAGCATTTTCTCTGC-3'	450	(31)
tdh[b]	Forward 5'-GTACCGATATTTTGCAAA-3'<br>Reverse 5'-ATGTTGAAGCTGTACTTGA-3'	382	(33)
trh[b]	Forward 5'-CTCTACTTTGCTTTCAGT-3'<br>Reverse 5'-TACGCTTATATAGGCGCTTA-3'	276	(34)
T3SS1	Forward5'-TCGGTTAGCGAAGGCGTA-3'<br>Reverse 5'-CCGCTGATAATGCCAGTA-3'	716	(16)
VP1680[c] (*vopQ*)	Forward 5'-CACTGATGGCGTATCTTGGTC-3'<br>Reverse 5'-GTCAAGCTGAGCGCCAG-3'	386	(35)
T3SS2α[c] (*vopP*)	Forward 5'-ATTCACCTTGACTTCTCTGC-3'<br>Reverse 5'-ACCTAAAGATACCGCTGCT-3'	379	(81)
T3SS2β[c] (*vopC*)	Forward 5'-AACCAACTTGCGACTAAATC-3'<br>Reverse 5'-TCCCGACAGTTTTTCTGCAC-3'	594	(32)

[a]species specific

ldh = lecithin dependent hemolysin

[b]virulence genes

tdh= themostable direct hemolysin

trh= themostable direct hemolysin-related hemolysin

[c]Type 3 secretion system genes

T3SS1 = Type 3 secretion system 1

T3SS2α = Type 3 secretion system 2α

T3SS2β = Type 3 secretion system 2β

Table S2 List and properties of 49 *V. parahaemolyticus* isolates from aquatic bird fecal samples

No.	Isolate name	Date of isolation	Virulence genes				
			tdh	*trh*	T3SS1	T3SS2α	T3SS2β
1	MUVP1	22/08/2016	-	-	+	-	-
2	MUVP2	22/08/2016	-	-	+	-	-
3	MUVP3	22/08/2016	-	-	+	-	-
4	MUVP4	5/9/2016	-	-	+	-	-
5	MUVP5	5/9/2016	-	-	+	-	-
6	MUVP6	5/9/2016	-	-	+	-	-
7	MUVP7	5/9/2016	-	-	+	-	-
8	MUVP8	5/9/2016	-	+	+	-	+
9	MUVP9	3/10/2016	-	+	+	-	+
10	MUVP10	17/10/2016	-	+	+	-	+
11	MUVP11	17/10/2016	-	+	+	-	+
12	MUVP12	31/10/2016	-	-	+	-	-
13	MUVP13	31/10/2016	-	-	+	-	-
14	MUVP14	31/10/2016	-	-	+	-	-
15	MUVP15	31/10/2016	-	-	+	-	-
16	MUVP16	30/01/2017	-	-	+	-	-
17	MUVP17	30/01/2017	-	-	+	-	-
18	MUVP18	14/02/2017	-	-	+	-	-
19	MUVP19	14/02/2017	-	-	+	-	-
20	MUVP20	13/03/2017	+	+	+	-	+
21	MUVP21	27/03/2017	-	-	+	-	-
22	MUVP22	27/03/2017	+	-	+	+	-
23	MUVP23	27/03/2017	+	-	+	+	-
24	MUVP24	27/03/2017	+	-	+	+	-
25	MUVP25	27/03/2017	-	-	+	-	-
26	MUVP26	14/11/2016	-	-	+	-	-
27	MUVP27	14/11/2016	-	-	+	-	-
28	MUVP28	14/11/2016	-	-	+	-	-
29	MUVP29	14/11/2016	-	-	+	-	-
30	MUVP30	28/11/2016	-	-	+	-	-
31	MUVP31	28/11/2016	-	-	+	-	-
32	MUVP32	13/12/2016	-	-	+	-	-
33	MUVP33	13/12/2016	-	-	+	-	-
34	MUVP34	13/12/2016	-	-	+	-	-
35	MUVP35	13/12/2016	-	-	+	-	-
36	MUVP36	30/01/2017	-	-	+	-	-

Table S2 List and properties of 49 *V. parahaemolyticus* isolates from aquatic bird fecal samples (cont.)

No.	Isolates name	Date of isolation	Virulence genes				
			tdh	*trh*	T3SS1	T3SS2α	T3SS2β
37	MUVP37	30/01/2017	-	-	+	-	-
38	MUVP38	30/01/2017	-	-	+	-	-
39	MUVP39	30/01/2017	-	-	+	-	-
40	MUVP40	14/02/2017	-	-	+	-	-
41	MUVP41	27/02/2017	-	-	+	-	-
42	MUVP42	13/03/2017	-	-	+	-	-
43	MUVP43	13/03/2017	-	-	+	-	-
44	MUVP45	28/04/2017	-	-	+	-	-
45	MUVP46	28/04/2017	-	-	+	-	-
46	MUVP47	28/04/2017	-	-	+	-	-
47	MUVP48	28/04/2017	-	-	+	-	-
48	MUVP49	28/04/2017	-	-	+	-	-
49	MUVP50	28/04/2017	-	-	+	-	-
